# Targeting filamin A reduces K-RAS–induced lung adenocarcinomas and endothelial response to tumor growth in mice

**DOI:** 10.1186/1476-4598-11-50

**Published:** 2012-08-02

**Authors:** Rajesh K Nallapalli, Mohamed X Ibrahim, Alex X Zhou, Sashidar Bandaru, Sai Naresh Sunkara, Björn Redfors, David Pazooki, Yin Zhang, Jan Borén, Yihai Cao, Martin O Bergo, Levent M Akyürek

**Affiliations:** 1 Institute of Biomedicine, Department of Medical Biochemistry and Cell Biology, University of Gothenburg, Sahlgrenska Academy, SE-405 30, Göteborg, Sweden; 2 Sahlgrenska Center for Cardiovascular and Metabolic Research, University of Gothenburg, Göteborg, Sweden; 3 Sahlgrenska Cancer Center, University of Gothenburg, Göteborg, Sweden; 4 Current address: Department of Medicine, Columbia University, New York, USA; 5 Department of Surgery, Institute of Clinical Sciences, University of Gothenburg, Göteborg, Sweden; 6 Department of Microbiology, Tumor and Cell Biology, Karolinska Institute, Stockholm, Sweden; 7 Department of Medicine and Health Sciences, Linköping University, Linköping, Sweden

**Keywords:** Cancer, Angiogenesis, Cytoskeleton, Migration

## Abstract

**Background:**

Many human cancer cells express filamin A (FLNA), an actin-binding structural protein that interacts with a diverse set of cell signaling proteins, but little is known about the biological importance of FLNA in tumor development. FLNA is also expressed in endothelial cells, which may be important for tumor angiogenesis. In this study, we defined the impact of targeting *Flna* in cancer and endothelial cells on the development of tumors* in vivo* and on the proliferation of fibroblasts *in vitro*.

**Methods:**

First, we used a *Cre*-adenovirus to simultaneously activate the expression of oncogenic K-RAS and inactivate the expression of *Flna* in the lung and in fibroblasts. Second, we subcutaneously injected mouse fibrosarcoma cells into mice lacking *Flna* in endothelial cells.

**Results:**

Knockout of *Flna* significantly reduced K-RAS–induced lung tumor formation and the proliferation of oncogenic K-RAS–expressing fibroblasts, and attenuated the activation of the downstream signaling molecules ERK and AKT. Genetic deletion of endothelial FLNA in mice did not impact cardiovascular development; however, knockout of *Flna* in endothelial cells reduced subcutaneous fibrosarcoma growth and vascularity within tumors.

**Conclusions:**

We conclude that FLNA is important for lung tumor growth and that endothelial *Flna* impacts local tumor growth. The data shed new light on the biological importance of FLNA and suggest that targeting this protein might be useful in cancer therapeutics.

## Background

Filamins are large actin-binding proteins that stabilize delicate three-dimensional actin networks and link them to cellular membranes during cell movements [1]. Filamins crosslink cortical filamentous actin into a dynamic orthogonal network and thereby confer membrane integrity and protection against mechanical stress. In addition to actin, filamins bind to numerous other proteins such as transmembrane receptors and signaling molecules and provide scaffolding functions and regulate multiple cellular behaviors [2]. Although filamins are classically known as cytoplasmic structural proteins, recent studies suggest that filamins are emerged as essential scaffolding proteins that play roles in cell signaling [2]. In addition, filamins interact with transcriptional factors to regulate their function and become members of transcriptional complex in the nucleus [2]. There are three members of the filamin family, filamin A (FLNA), filamin B (FLNB) and filamin C (FLNC). Both human *FLNA* and mouse filamin A (*Flna)* genes are located on the X chromosome. During embryogenesis as well as in adults, FLNA is the most abundant isoform, is ubiquitously expressed throughout the body and appears to be the major filamin responsible for cardiovascular development.

Many studies have reported increased expression of FLNA in human cancer tissues such as hepatic [3], breast, and astrocytoma [4] as well as in different cancer cell lines and human lung cells [5]. FLNA may mediate the effects of signaling pathways on both cancer and endothelial cell motility during tumorigenesis. In addition, the RAS-signaling pathway has attracted considerable attention as a target for anticancer therapy because of its important role in carcinogenesis [6]. Interestingly, in mammalian cells, the generation of actin-based dynamic motile structures is regulated by small GTPases of the Rho family and FLNA interacts with these GTPases [7]. Following integrin binding to extracellular matrix ligands, small GTPases are activated, leading to actin polymerization and the formation of lamellipodia and filopodia. Branched actin networks are particularly important for the formation of lamellipodia that are believed to be the actual motors that pull cells forward. Filopodia originate from the pre-existing lamellipodial actin network that is prevented from capping and, as a result, can elongate at the leading edge of the lamellipodia. Mutations in the K-RAS gene render the protein unable to hydrolyze GTP and have been found in 20–30% of non-small-cell lung cancers [8]. The small GTP-binding proteins K-RAS, H-RAS and N-RAS belong to a family of oncoproteins associated with many types of other human cancer. The *K-RAS* gene is designated *Kras2* in the mouse. RAS proteins interact with a number of effector proteins that in turn activate important signaling pathways, including the RAF/MEK/ERK and the PI3K/PKB/AKT pathways [8]. The complexity of the RAS signaling pathway and the difficulty of targeting the RAS protein itself necessitate continuous searches for additional mechanisms that regulate RAS-induced tumor growth.

A recent study showed that an interaction between active RAS and FLNA is responsible for maintaining endothelial barrier function [9]. Loss of the RAS-FLNA interaction promotes VE-Cadherin phosphorylation and changes in downstream effectors that lead to endothelial leakiness. Interestingly, complete *Flna* deficiency results in embryonic lethality in mice due to severe cardiac structural malformations [10]. In addition, it has been reported that breakdown of the endothelial lining could weaken the blood vasculature, leading to vascular abnormalities [10].

Despite the many studies focusing on the expression and function of FLNA in tumor cells, its role in endothelial cells and cell migration, very little is known about the importance of FLNA in endogenous tumor growth. In addition, the specific role of FLNA in oncogenic angiogenesis has not yet been explored. In this study, we used two different tumor models in mice to determine the role of FLNA in K-RAS–induced lung tumor formation and the role of endothelial FLNA during tumor growth.

## Methods

### Mice

All mice included in this study had a C57Bl/6 genetic background. Male heterozygous mice containing a floxed stop codon (LSL) before the constitutively active *Kras2* promoter (*Kras2*^LSL^) [11,12] were bred with female mice homozygous for a conditional “floxed” allele of *Flna* (*Flna*^fl/fl^) [10]. For further experiments, female mice expressing *Flna*^fl/fl^ were also bred with male transgenic mice expressing Cre under control of the *VE-Cadherin* promoter, an endothelial cell-specific promoter (The Jackson Laboratory, Bar Harbor, ME). Because the *Flna* gene is located on the X chromosome, hemizygous male mice were designated *Flna*^o/fl^. Mouse experiments were approved by the Research Animal Ethics Committee at the University of Gothenburg.

### Genotyping

Genomic DNA was isolated from mouse tail biopsies. The *Flna* allele was PCR amplified with forward (*F*) primer 5`-TCTTCCTCTTTCAGCTGG-3`and reverse (*R*) primer 5`-CACAGTCACCTGTTCCCA-3`, yielding 280-bp “floxed” and 250-bp wild-type fragments. The *Kras2*^LSL^ allele was genotyped by PCR amplification of genomic DNA as described [13] and yielded a 600-bp fragment. The activated *Kras2*^G12D^ allele was genotyped as described [13] and yielded 320-bp activated and 280-bp wild-type fragments.

### Inhalation of Cre-adenoviral vector

The *Cre*-adenovirus was prepared as a calcium-phosphate coprecipitate and incubated for 20 min at room temperature as described [13]. Groups of *Flna*^o/fl^*Kras2*^LSL/+^ (n = 6) and littermate control *Flna*^o/+^*Kras2*^LSL/+^ (n = 6) mice at the age of 4–5 weeks were allowed to inhale 125 μl of virus at a concentration of 5 × 10^7^ PFU during light isoflurane anesthesia as described [11]. Additionally, *Flna*^o/fl^ alone (n = 7) and wild-type C57Bl/6 (n = 7) male mice were included as controls. The mice were euthanized 12 weeks after inhalation and lungs were harvested for tumor analysis. All experimental mice were similar in body weight before and after infection with Ad-Cre at the age of 4 weeks (11.1 ± 0.5 g) and 16 weeks (32.7 ± 1.1 g).

### Histological analysis of lung tumors

Lungs from *Flna*^o/+^*Kras2*^LSL/+^ and *Flna*^o/fl^*Kras2*^LSL/+^ mice were inflated and fixed with paraformaldehyde. Each lobe was separately embedded in paraffin as described earlier [12]. Five sections at 200 μm intervals of lobes 3 and 5 were stained with hematoxylin and eosin (H&E). To quantify lung tumor area, entire lung sections at 5 different levels in each lobe were captured under the microscope at magnification × 5 prior to being viewed with a Zeiss epifluorescence microscope. Images were obtained and digitized (TIFF format; with a resolution of 8 pixels/μm). H&E stained color was picked by Biopix software (Version 2.1.3; Biopix, http://www.biopix.se) according to the hue, saturation and brightness of the color and dense tumor area was assigned with an artificial yellow color. Normal pulmonary and non-stained airway structures were assigned with an artificial blue color. The same program setting was applied to all images and areas measured automatically by the software. To study the expression of FLNA in lung tumors, sections were fixed in 4% PFA for 24 h, embedded in paraffin, sectioned to 5 μm thickness and immunohistochemically stained using an anti-FLNA antibody (Chemicon International) as described previously [14]. Appropriate IgG controls and omission of primary antibody served as negative controls to immunohistochemistry. To quantify tumor endothelial cell density, lungs were used for immunohistochemistry as described previously [15]. Sections were stained with a primary antibody, rat monoclonal anti-CD31 (PECAM-1) (Pharmingen). Secondary FITC-conjugated and Alex-conjugated antibodies (1:100; Molecular Probes, Sweden) were applied. Sections were then washed and mounted with mounting media (Dako) and analyzed using a ZEISS Axioskop II microscope. The capillary density within the tumors was calculated by computer in five randomly selected areas captured from PECAM-stained lung sections.

### Isolation of mouse embryonic fibroblasts

*Kras2*^LSL/+^ male mice were bred with *Flna*^fl/fl^ females. Embryos were harvested from pregnant females at embryonic day 14. Genomic DNA from embryos was genotyped for both *Kras2*^LSL^ and *Flna*^fl^ alleles. Isolation and immortalization of mouse embryonic fibroblasts (MEF) were performed as described [16]. Embryonic liver, intestines, head and extremities were removed, the rest was tweezed on a petri plate with trypsin-EDTA and shaken on a rocking platform at 4°C overnight. The next day, 5 ml of prewarmed MEF medium was added, mixed by pipetting up and down and incubated for 5 minutes. Supernatant containing cells were collected and added to T175 flask containing 20 ml of MEF medium, in which DMEM was supplemented with 10% FBS, 1% penicillin/streptomycin, 1% NEAA and 1% glutamine.

### Proliferation assay

30 × 10^3^ MEFs were seeded in triplicate in 6-well plates and incubated in serum-free medium overnight. The medium was then replaced with medium containing 10% serum and the cells were trypsinized and counted at 1, 2, 3 and 4 days of observation. Results of triplicate experiments are given as mean ± SD values and presented as fold-increases normalized to day 1.

### Western blotting

Equal amounts of protein from total MEF extracts were size-fractionated on 4–12% SDS/PAGE gels (Invitrogen). The proteins were transferred to nitrocellulose membranes, blocked with 5% milk and incubated with antibodies recognizing FLNA (Bethyl Laboratories), Actin (Sigma), p-ERK, total ERK, p-AKT, and total AKT (Cell Signaling Technology) as described earlier [14]. Protein bands were visualized with a horseradish peroxidase-conjugated secondary antibody (Cell Signaling Technology) and the ECL Western Blotting System (GE Healthcare). Images of immunoblots were captured using a gel documentation system (Bio-Rad, Gel Doc 2000). Protein bands from triplicate experiments were analyzed by densitometry with Quantity One 4.4.0 software (Bio-Rad).

### Isolation of cardiac and pulmonary endothelial cells and RT-PCR

Whole mouse hearts or lungs were placed in ice-cold PBS, minced into 1 mm^3^ pieces and digested using 100 μg/ml Collagenase type III (Sigma) in Hanks’ balanced salt solution (HBSS, Invitrogen) supplemented with 1% BSA and 100 U/ml DNase at 37°C for 15 min with gentle agitation as described earlier [17]. Tissues were then gently pressed through 100 μm and then 40 μm cell strainers (Falcon, BD Biosciences). Cells were washed out from the strainer in 2 ml of HBSS supplemented with 1% BSA and 100 U/ml DNase, pelleted at 200 × g for 5 min, suspended in 1.5 ml HBSS supplemented with 1% BSA and 100 U/ml DNase, and again pelleted and resuspended. Rat anti-CD31 (BD Pharmingen) antibody-coated magnetic beads (Dynabeads M-450, sheep anti-Rat IgG, Dynal) were added, and after incubation at 4°C for 30 min with gentle agitation, pulmonary endothelial cells were isolated with a magnetic particle concentrator (MPC, Dynal) and washed three times with HBSS supplemented with 1% BSA.

The purity of the isolated endothelial cells was confirmed by RT-PCR using primers to detect mRNA expression of mouse *Pecam* as a positive control and primers to amplify the smooth muscle-specific mRNA expression of mouse *Sm22α* as a negative control. Mouse primers amplifying transcripts of *Flna* (*F* primer 5'-AAGCCCTCTGCAGTTCTATGTTGA T-3' and *R* primer 5'-GCA AACGTTTCAGCAGACAGGGTT-3'), *18 S* (*F* primer 5'-AGATCAAAACCAACCCGGTGA-3' and *R* primer 5'-GGTAAGAGCATCGAGGGGGC-3'), *Pecam* (*F* primer 5'-AGGGGACCAGCTGCACATTAG G-3' and *R* primer 5'-AGGCCGCTTCTCTTGACCACT T-3') and *SM22α* (*F* primer 5'-CCACACTCTATACTTTAGCTCTGCCTCAAC-3' and *R* primer 5'-CAGGCTGTTCACCAATTTGCTCAGAAT-3') were used for RT-PCR as described [16].

### Histological analysis of hearts

Hearts were removed from adult *VE-Cad*^Cre/+^*Flna*^o/+^ and *VE-Cad*^Cre/+^*Flna*^o/fl^ mice, fixed with paraformaldehyde, embedded in paraffin, sectioned, and stained with H&E to study histomorphology.

### Analysis of cardiac function by echocardiography

Transthoracic color Doppler echocardiography was performed on isoflurane-anesthetized mice at the age of 8 weeks as described [15]. During the procedure, the mice were maintained lightly anesthetized with an isoflurane dose of approximately 1.2% in air using a nose mask. Examinations were performed using a high-frequency 15-MHz linear transducer (CL 15–7; Philip Medical System) connected to an HDI 5000 ultrasound system (ATL; Philip Medical System). All measurements were based on the average of at least 3 cardiac cycles. Fractional shortening was calculated using left ventricular (LV) end diastolic diameter (LVEDd) and systolic diameter (LVESd) values as (LVEDd–LVESd)/LVEDd × 100. Other measurements of cardiac function included diastolic and systolic LV internal diameter, diastolic posterior wall thickness, relative wall thickness, and LV mass by M-mode as described earlier [15].

### Migration assay

Migration assays were performed on lung endothelial cells in passage 2–3. Cell migration was quantified with a modified Boyden chamber assay [16] where the ability of cells to migrate through a micropore nitrocellulose filter (8 μm thick, 8 μm pores in diameter) was measured. Approximately 3 × 10^4^ cells were seeded in the upper chamber in medium containing 2% FBS; the chamber was lowered into a well containing 10% FBS. After 4 h of incubation at 37°C, migrated cells were fixed to the filter in methanol and stained with Giemsa. All cells that had migrated through the filter were counted using a light microscope. Triplicates of each sample are presented as mean ± SD values.

### Subcutaneous tumor cell inoculation in mice

Five-week-old *VE-Cad*^Cre/+^*Flna*^o/fl^ and control *VE-Cad*^Cre/+^*Flna*^o/+^ male mice were given subcutaneous injections of 1 × 10^6^ of T241 mouse fibrosarcoma or B16 melanoma cells in the dorsal back region as described earlier [18]. Tumor volume was calculated by measurements of width^2^ ×length × 0.52. The diameter of isolated tumors was measured every second day starting one week after inoculation and tumors were harvested before they reached a size of 1.5 cm^3^. Tumors were carefully resected, weighed, placed in OCT, snap-frozen in liquid nitrogen and stored at −80°C. The vascular network within the fibrosarcomas was visualized by whole-mount immunostaining of vascular endothelial cells and pericytes using anti-PECAM and anti-NG-2 antibodies, respectively, as described earlier [18].

### Statistical analysis

Data are given as mean ± standard deviation (SD). Differences between experiment groups were analyzed for statistical significance (*P* < 0.05) by two-way ANOVA or Student’s *t* test.

## Results

### Targeting *Flna* reduces K-RAS–induced lung tumor development in mice

**Figure 1:**
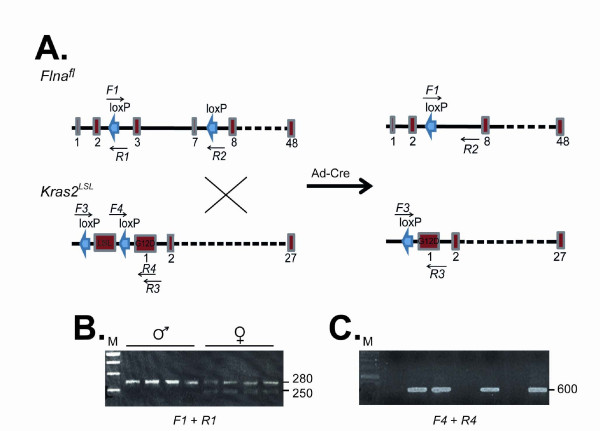
**Strategy for inactivating the expression of FLNA and simultaneously activating the expression of K-RAS**^**G12D**^** in mouse lungs. (A)** Schematic illustration of mouse allele, *Flna*^fl^ and *Kras2*^LSL^. Numbered boxes represent exons. Infection of lungs with adenoviral vector encoding Cre (Ad-Cre) yields a truncation of exons 2–8 of *Flna* and removal of LSL before constitutive activation of the *Kras2* promoter. Sites for forward (*F*) and reverse (*R*) oligonucleotide primers are indicated (arrows). **(B)** Genotyping of male (*Flna*^o/fl^) and female (*Flna*^+/fl^) mouse tails by genomic PCR using *F1* and *R1* primers detecting *Flna* alleles. Higher band represents *Flna*^fl^ allele. **(C)** Genotyping of mouse tails by genomic PCR using *F4* and *R4* primers for *LSL*. Amplicons represent *Kras2*^LSL^.

**Figure 2:**
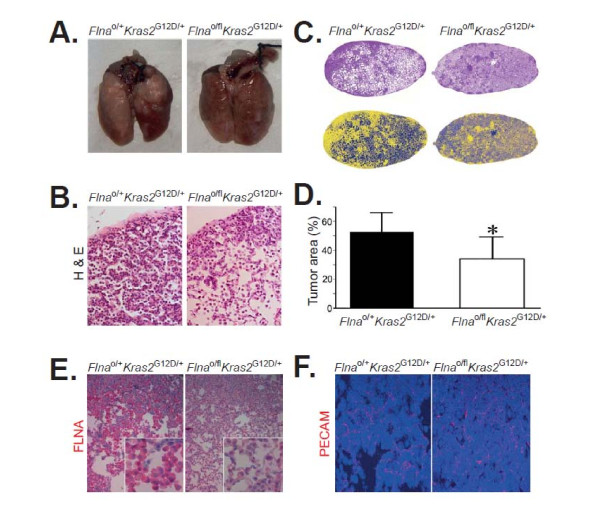
**Inactivation of *Flna* reduces tumor burden in a *Kras2* **^**G12D**^**-induced lung cancer in mice. (A)** Photographs of lungs 12 weeks after inhalation of adenoviral vector encoding Cre (Ad-Cre). Note the extensive tumor lesions (white areas) on the surface of lungs from *Flna*^o/+^*Kras2*^G12D/+^ male mice and the relatively normal appearance of the lungs from *Flna*^o/fl^*Kras2*^G12D/+^ male mice. **(B)** Hematoxylin & Eosin (H&E)-stained sections of representative lesions in lungs from *Flna*^o/+^*Kras2*^G12D/+^ and *Flna*^o/fl^*Kras2*^G12D/+^ male mice. Original magnifications × 20. **(C)** Computer-assisted image analysis of H&E-stained lung tumor areas (upper panels) using artificial colors (lower panels). Yellow color represents lung tumor areas, whereas blue color indicates normal pulmonary structures and airways. Original magnifications × 5. **(D)** Mean ± SD values of percentage of lung tumor areas from entire lower right and left lung lobes sectioned at five different levels. Each group consisted of at least 5 mice. Student’s *t* test, *P < 0.05. **(E)** Immunohistochemical staining of *Flna*^o/+^*Kras2*^G12D/+^ and *Flna*^o/fl^*Kras2*^G12D/+^ lung tumor areas using an anti-FLNA antibody. Red color represents immunohistochemical FLNA-positivity, whereas blue color indicates nuclear counterstaining. Original magnifications × 20 and × 100 (insets). **(F)** Immunofluorescence detecting of endothelial cells in lung tumors by an antibody against PECAM (red). Nuclear DAPI staining (blue). Original magnifications × 20.

To study the role of *Flna* in K-RAS–induced tumor development, we bred “floxed” conditional *Flna* knockout mice (*Flna*^fl^) with mice harboring a *loxP*-STOP-*loxP* K-RAS^G12D^ allele (*Kras2*^LSL/+^) (Figure 1A). The genotype of offspring was confirmed by genomic PCR for *Flna*^fl^ (Figure 1B) and *Kras2*^LSL^ (Figure 1C). *Flna* is located on the X chromosome and we performed experiments with male *Flna*^o/fl^*Kras2*^LSL/+^ and male littermate control *Flna*^o/+^*Kras2*^LSL/+^ mice. We administered a *Cre*-adenovirus to 4–5-week-old mice by intranasal instillation. *Cre* expression activated K-RAS^G12D^ expression in the lung of both groups of mice and simultaneously inactivated the single copy of *Flna* in *Flna*^o/fl^*Kras2*^LSL/+^ mice. No experimental complication such as mouse death due to adenoviral inhalation was seen. After 12 weeks, mice were euthanized and lungs harvested. C57Bl/6 wild-type and *Flna*^o/fl^ alone control mice did not develop lung tumors after inhalation of Ad-Cre and were, therefore, not further processed for histological analysis. Both *Flna*^o/fl^*Kras2*^LSL/+^ and *Flna*^o/+^*Kras2*^LSL/+^ mice developed macroscopically visible lung tumors (Figure 2A). However, macroscopic evaluation revealed a reduced tumor burden in *Flna*^o/fl^*Kras2*^LSL/+^ compared to *Flna*^o/+^*Kras2*^LSL/+^ mice. Histological examination of lung sections showed pulmonary adenocarcinomas (Figure 2B). The tumor area in lungs of *Flna*^o/fl^*Kras2*^LSL/+^ mice was reduced by 35% compared to *Flna*^o/+^*Kras2*^LSL/+^ (Figure 2C and D, *P* < 0.05; 34.1 ± 15.0% *versus* 52.3 ± 13.3%). Less intense immunohistochemical expression of FLNA was observed in *Flna*^o/fl^*Kras2*^LSL/+^ lung tumors compared to *Flna*^o/+^*Kras2*^LSL/+^ lung tumors as a result of *Flna* deletion in pulmonary epithelial cells after infection with Ad-Cre (Figure 2E). There were similar numbers of vascular structures in both groups of mice as quantified by immunofluorescence staining with anti-PECAM antibodies (Figure 2F).

### *Flna* deficiency reduces proliferation of mouse embryonic fibroblasts

**Figure 3:**
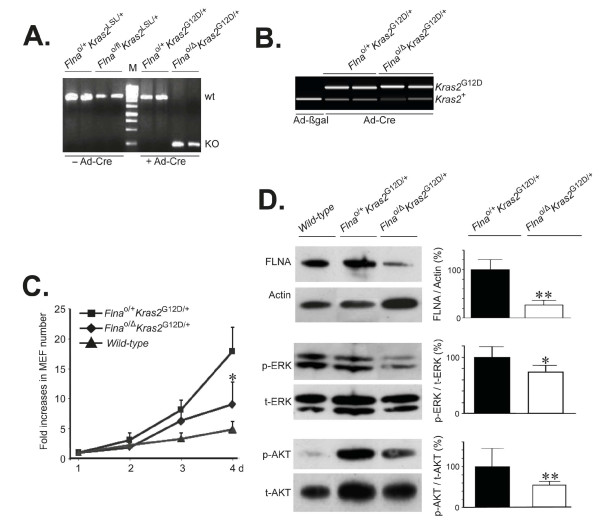
**Inactivation of *Flna* reduces proliferation of K-RAS**^**G12D**^**–expressing primary mouse embryonic fibroblasts (MEF). (A)** Genotyping of MEF by genomic PCR using *F1* and *R2* primers, as indicated in Figure 1, for *Flna* before (left lanes) and after (right lanes) infection with adenoviral vector encoding *Cre* (Ad-Cre). M, DNA ladder. **(B)** Genotyping of MEF by genomic PCR using *F4* and *R4* primers, as indicated in Figure 1, for *Kras2* after infection with Ad-Cre. Upper band represents insertion of a single *loxP* site after removal of *LSL* in the *Kras2* gene (*Kras2*^G12D^). **(C)** Proliferation assay of K-RAS–induced MEF with or without *Flna* up to 4 days. Wild-type MEF cells were included as controls. **(D)** Representative immunoblots of FLNA, phosphorylated ERK (p-ERK) and AKT (p-AKT) in K-RAS–induced cells with or without *Flna* after infection with Ad-Cre. Actin loadings and total ERK (t-ERK) and AKT (t-AKT) immunoblots served as internal controls. Mean ± SD values of densitometric readings of band intensities in triplicate experiments. Student’s *t* test, *P < 0.05, **P < 0.01.

We isolated fibroblasts from *Flna*^o/fl^*Kras2*^LSL/+^ and *Flna*^o/+^*Kras2*^LSL/+^ embryos and incubated them with the *Cre-*adenovirus to activate K-RAS^G12D^ expression in cells of both genotypes and simultaneously inactivate the single *Flna* copy in *Flna*^o/fl^*Kras2*^LSL/+^ cells. The genotypes of the resulting cells were *Flna*^o/Δ^*Kras2*^G12D/+^ and *Flna*^o/+^*Kras2*^G12D/+^ (Figure 3A and B). As expected, the proliferation of *Flna*^o/+^*Kras2*^G12D/+^ cells increased compared to wild-type cells due to K-RAS^G12D^ expression (Figure 3C). However, the proliferation of *Flna*^o/Δ^*Kras2*^G12D/+^ cells was reduced compared to *Flna*^o/+^*Kras2*^G12D/+^ cells (Figure 3C) and this reached significance 4 days after infection with *Cre*-adenoviral vector (*P* < 0.05, 8.46 ± 0.64 *versus* 17.4 ± 5.05 fold increases in cell number). The total levels of both ERK and AKT were smaller in wild-type control cells than *Flna*^o/+^*Kras2*^G12D/+^ cells (Figure 3D, immunoblots). Compared to *Flna*^o/+^*Kras2*^G12D/+^ cells, levels of FLNA protein in *Flna*^o/Δ^*Kras2*^G12D/+^ cells were reduced by 74% as quantified by Western blotting of whole-cell lysates (Figure 3D). Interestingly, stead-state levels of phospho-ERK and phospho-AKT in *Flna*-deficient cells were reduced by 25 and 55%, respectively, compared to controls (Figure 3D).

### *Flna* deficiency in endothelial cells has no apparent impact on cardiac development and function

**Figure 4:**
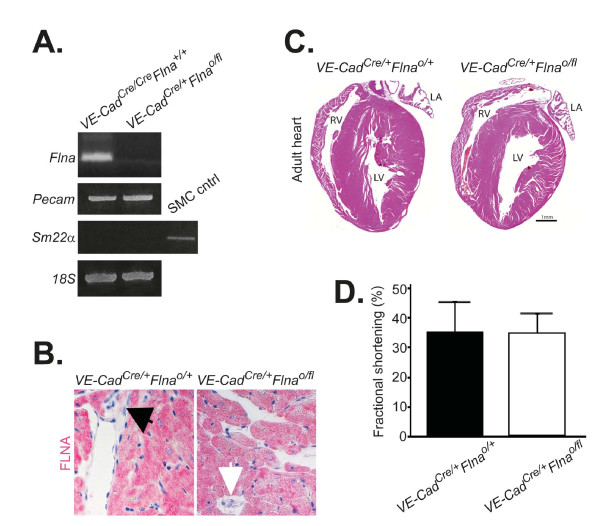
**Normal cardiac morphology and function in mice deficient for FLNA in vascular endothelium. (A)** RT-PCR analysis of *Flna* and *Pecam* transcripts in cardiac endothelial cells extracted from a female mouse encoding Cre under the *VE-Cadherin* promoter and expressing wild-type *Flna* (*VE-Cad*^Cre/Cre^*Flna*^+/+^) and from a male mouse that expresses no FLNA (*VE-Cad*^Cre/+^*Flna*^o/fl^). *Sm22α* served as a negative control to demonstrate the purity of endothelial cell extraction from vascular smooth muscle cells and *18 S* served as internal loading control. **(B)** Immunohistochemical detection of FLNA in hearts. Red color represents immunohistochemical positivity and blue color indicates nuclear counterstaining. The black arrow points to a FLNA-positive endothelial cell in *VE-Cad*^Cre/Cre^*Flna*^o/+^ heart, whereas the white arrow indicates a FLNA-negative endothelial cell in *VE-Cad*^Cre/+^*Flna*^o/fl^ heart. Original magnifications × 20. **(C)** No histomorphological differences in adult cardiac sections between *VE-Cad*^Cre/+^*Flna*^o/+^ and *VE-Cad*^Cre/+^*Flna*^o/fl^ male mice. Hematoxylin & Eosin stain. LA; left atrium, RV; right ventricle, LV; left ventricle. **(D)** No differences in percentage of cardiac fractional shortening between *VE-Cad*^Cre/+^*Flna*^o/+^ and *VE-Cad*^Cre/+^*Flna*^o/fl^ male mice as measured by cardiac ultrasound.

To define the importance of endothelial FLNA in cardiovascular development, we bred *Flna*^o/fl^mice with mice harboring a Cre transgene driven by the endothelial cell-specific VE-Cadherin promoter. We isolated endothelial cells from hearts of *VE-Cad*^Cre/+^*Flna*^o/fl^ and littermate control *VE-Cad*^Cre/+^*Flna*^o/+^ mice with PECAM-conjugated microbeads. The purity of the endothelial cell preparation was confirmed by the presence of *Pecam* transcripts and by the absence of *Sm22α* transcripts. *Flna* mRNA transcript and protein were essentially eliminated in *VE-Cad*^Cre/+^*Flna*^o/fl^ endothelial cells (Figure 4A). To assess the impact of endothelial cell-specific inactivation of FLNA on cardiac morphology, we performed histological staining (Figure 4B). Additionally, cardiac tissues with or without endothelial FLNA (*VE-Cad*^Cre/+^*Flna*^o/+^ and* VE-Cad*^Cre/+^*Flna*^o/fl^, respectively) were immunohistochemically stained (Figure 4C). Finally, we performed echocardiography analyses to measure the percentage of cardiac fractional shortening (Figure 4D). These studies indicated that cardiac histomorphology and cardiac pump function were similar in control *VE-Cad*^Cre/+^*Flna*^o/+^ and *VE-Cad*^Cre/+^*Flna*^o/fl^ mice. Other parameters of cardiac function were also found to be similar between *VE-Cad*^Cre/+^*Flna*^o/+^ and *VE-Cad*^Cre/+^*Flna*^o/fl^ hearts, including left ventricular internal diameter diastole (4.39 ± 0.50 mm *versus* 4.18 ± 0.26 mm, *P* = 0.50 at baseline and 3.64 ± 0.54 mm *versus* 3.57 ± 0.19 mm, *P* = 0.83 during dobutamine stress), left ventricular internal diameter systole (3.04 ± 0.76 mm *versus* 2.62 ± 0.50 mm, *P* = 0.39 at baseline and 1.96 ± 0.42 mm *versus* 1.68 ± 0.24 mm, *P* = 0.30 during dobutamine stress), posterior diastolic wall thickness (0.80 ± 0.11 mm *versus* 0.79 ± 0.08 mm, *P* = 0.8), relative diastolic wall thickness (0.18 ± 0.01 mm *versus* 0.19 ± 0.02 mm, *P* = 0.71), and left ventricular mass calculated by M-mode echo (142.37 ± 51.66 mg *versus* 124.33 ± 22.65 mg, *P* = 0.55). Taken together, these data suggest that FLNA in endothelial cells is dispensable for heart development and function.

### *Flna* deficiency in endothelial cells reduces migration and the ability to support subcutaneous tumor growth

**Figure 5:**
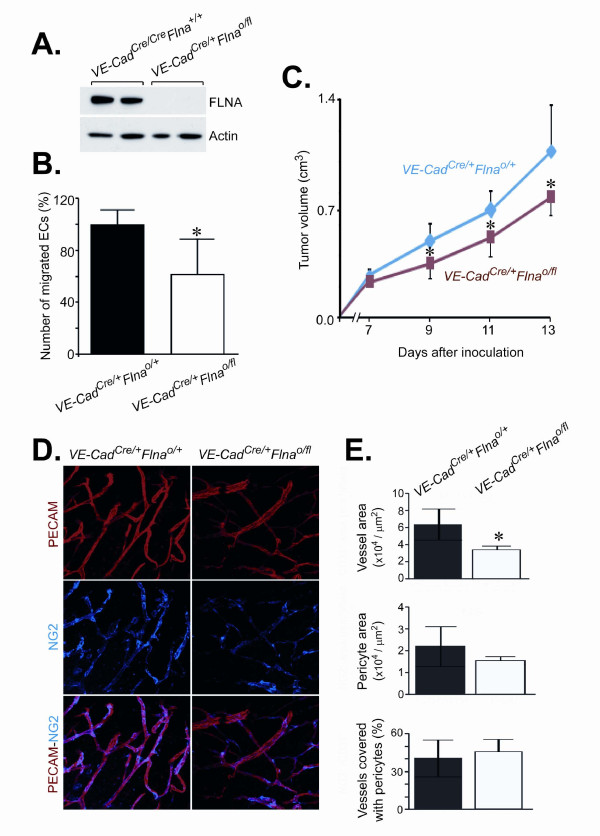
**Fibrosarcoma cells inoculated into mice lacking *Flna* in endothelial cells form smaller tumors. (A)** Immunoblot of endothelial cells extracted from *VE-Cad*^Cre/+^*Flna*^o/+^ and *VE-Cad*^Cre/+^*Flna*^o/fl^ lungs. Actin loading served as internal loading. **(B)** Decreased percentage of migrated *VE-Cad*^Cre/+^*Flna*^o/fl^ endothelial cells as assayed by a modified Boyden’s chamber. Student’s t test, *P < 0.05. **(C)** Tumor size measurements up to 13 days after inoculation of mouse fibrosarcoma cells into *VE*-*Cad*^Cre/+^*Flna*^o/+^ and *VE*-*Cad*^Cre/+^*Flna*^o/fl^ male mice. Student’s t test, *P < 0.05. **(D)** Representative micrographs of vascular networks in *VE*-*Cad*^Cre/+^*Flna*^o/+^ and *VE*-*Cad*^Cre/+^*Flna*^o/fl^ fibrosarcomas. Red color represents PECAM-positive endothelial cells, whereas blue color identifies NG-2–positive pericytes. Original magnifications × 20. (E) Quantification of microvascular and pericyte areas, and percentage of pericyte-covered vascular structures within tumors. Student’s t test, *P < 0.05.

To define the ability of FLNA in endothelial cells to support tumor growth, we bred *Flna*^o/fl^mice with mice harboring a Cre transgene driven by the endothelial cell-specific VE-Cadherin promoter. We isolated lung endothelial cell proteins from *VE-Cad*^Cre/+^*Flna*^o/fl^ and littermate control *VE-Cad*^Cre/+^*Flna*^o/+^mice with PECAM-conjugated microbeads. This indicated that FLNA protein was essentially eliminated in *VE-Cad*^Cre/+^*Flna*^o/fl^ endothelial cells (Figue 5A). Interestingly, the ability of *Flna*-deficient endothelial cells to migrate was reduced by 38% compared to control cells (224 ± 25 *VE-Cad*^Cre/+^*Flna*^o/+^ cells *versus* 139 ± 36 *VE-Cad*^Cre/+^*Flna*^o/fl^, *P* < 0.05) (Figure 5B). We then subcutaneously inoculated T241 mouse fibrosarcoma cells into *VE-Cad*^Cre/+^*Flna*^o/fl^ and *VE-Cad*^Cre/+^*Flna*^o/+^ mice and measured tumor volume (Figure 5C). The subcutaneous tumors grew significantly slower in *VE-Cad*^Cre/+^*Flna*^o/fl^ compared to *VE-Cad*^Cre/+^*Flna*^o/+^ mice and were 0.77 ± 0.12 cm^3^ (n = 7) and 1.06 ± 0.77 cm^3^ (n = 8), respectively, at 13 days after inoculation (*P* < 0.05). Similarly, inoculation of B16 mouse melanoma cells resulted in reduced tumor growth. Melanoma tumor volumes in *VE-Cad*^Cre/+^*Flna*^o/fl^ and *VE-Cad*^Cre/+^*Flna*^o/+^ mice were 0.22 ± 0.05 cm^3^ (n = 6) and 0.32 ± 0.09 cm^3^ (n = 7), respectively, at day 9 (*P* < 0.05) and 0.34 ± 0.05 cm^3^ and 0.47 ± 0.12 cm^3^, respectively, at day 11 (*P* < 0.01) (data not shown). To visualize the vascular network within fibrosarcomas inoculated into *VE-Cad*^Cre/+^*Flna*^o/fl^ and *VE-Cad*^Cre/+^*Flna*^o/+^ mice, whole-mounted tumor tissues were stained with antibodies detecting endothelial cells (PECAM) and pericytes (NG2) (Figure 5D). Quantification of these analyses indicated that the vascular area within the fibrosarcomas was significantly reduced in *VE-Cad*^Cre/+^*Flna*^o/fl^ mice compared to *VE-Cad*^Cre/+^*Flna*^o/+^ mice (6.38 ± 1.82 × 10^4^/ μm^2^* versus* 3.47 ± 0.40 × 10^4^/ μm^2^, *P* = 0.002) (Figure 5E, upper panel). However, the number of pericytes (2.21 ± 0.89 × 10^4^/ μm^2^*versus* 1.56 ± 0.16 × 10^4^/ μm^2^) or pericyte-covered vascular areas (41 ± 15% *versus* 46 ± 10%) was not changed (Figure 5E, middle and lower panels).

## Discussions

In this study, we demonstrated that knockout of *Flna* reduces K-RAS–induced lung tumor development *in vivo* and reduces the proliferation of K-RAS^G12D^–expressing fibroblasts *in vitro*. The reduced fibroblast proliferation was associated with reduced levels of activated ERK and AKT. Moreover, we showed that targeting *Flna* specifically to endothelial cells reduces their migratory ability and retards local tumor growth.

Endogenous activation of K-RAS^G12D^ in the lung results in adenocarcinoma that originates in terminal and respiratory bronchi or in the alveolar epithelium [12]. Adenocarcinoma of the lung is the most common type of lung cancer in lifelong non-smokers [19]. Studies of human lung cancer have shown that adenocarcinoma is the only subtype associated with RAS mutations [20]. In the *Kras2*^LSL^ model, inhalation of *Cre*-adenoviral vector results primarily in infection of respiratory epithelial cells, where Cre deletes the “floxed” stop cassette to activate the expression of K-RAS^G12D^ from the endogenous promoter. In the *Flna*^o/fl^*Kras2*^LSL/+^ mice, Cre expression simultaneously inactivated *Flna*. Although *Flna* deficiency significantly reduced lung tumor development, it did not abolish tumors. This could conceivably be caused by partial recombination, where the K-RAS allele would be activated by Cre but *Flna* would not be inactivated in every cell as reported [12,19]. We believe that Cre-adenovirus infection in the *Kras2*^*LSL*^ lung does not result in Cre expression and recombination of “floxed” alleles in endothelial cells as these cells are located at some distance from the respiratory epithelium.

The RAS/RAF/MEK/ERK and RAS/PI3K/PTEN/AKT signaling pathways are cascades regulated by phosphorylation and dephosphorylation by specific kinases, phosphatases, as well as GTP/GDP exchange proteins, adaptor proteins and scaffolding proteins [21]. These pathways play key roles in the proliferation of tumor cells and growth of tumors. Therefore, inhibitors targeting these pathways have many potential uses for suppression of cancer. However, cancer therapy is often complex as there are relatively few cancers which proliferate in response to a single molecule interaction which prevents them from being treated with a monospecific drug. Therefore, new targets need to be identified to develop more effective treatments. In this study, we showed that cellular K-RAS–induced proliferation was reduced by *Flna* deficiency. The effect was associated with impaired activation of the RAS downstream effectors ERK and AKT. This finding suggests that targeting FLNA might be considered in combined treatment with established targets. The impact of *Flna* deficiency on cellular proliferation has not been reported in normal MEFs [10], however, we observed that *Flna*-deficiency impairs proliferation of these cells when induced by K-RAS.

What is the mechanism behind the reduced tumor growth and proliferation in K-RAS–expressing cells? One potential explanation is that FLNA acts as a scaffolding protein and is required for efficient spatial and temporal activation of effectors in the RAS pathway and that the absence of FLNA directly affects RAS signaling. Another is that FLNA is involved in regulating the dynamics of the actin cytoskeleton and that the impact of *Flna* deficiency on tumor growth reflects a more general role for the protein in cellular structure and function. A third, perhaps more intriguing possibility, is that the FLNA protein can be cleaved in the hinge region and regulate gene transcription in the nucleus [22]. Over the next few years, studies will likely shed light on these different possibilities.

Because FLNA has been shown to be important in vascular cells [10,23], we were interested in defining the impact of *Flna* deficiency on both normal and tumor endothelial cells. Interestingly, mice lacking *Flna* in endothelial cells had no apparent phenotypes. Cardiac development and function appeared to be normal and vascular integrity was unaffected. This finding was surprising for several reasons. First, *Flna*-deficient mice showed prominent cardiovascular abnormalities as well as extensive defects in cell–cell junctions that were particularly prominent in vascular endothelial cells [10]. And second, multiple functions of FLNA in endothelial cells have recently been reported including caveolae internalization and trafficking [24] and chemotaxis [25]. Interestingly, a crucial role for another filamin, FLNB, in endothelial cell migration and in the angiogenic process in adult endothelial cells has been reported [26]. As both filamin genes are highly conserved and the filamin proteins exhibit high amino acid identity and can also form heterodimers [2], it is likely that FLNA and FLNB have both unique and overlapping roles in the vascular endothelium. Regardless, our findings suggest that FLNA might not be as important for endothelial cell function as had previously been appreciated. We did, however, observe reduced migration of *Flna*-deficient endothelial cells. Moreover, we found that fibrosarcoma and melanoma tumor growth under the skin of mice lacking *Flna* in endothelial cells was reduced. As FLNA was specifically deficient in vascular endothelial cells, we observed a significantly reduced number of vascular endothelial cells, but not pericytes. This result suggests that endothelial FLNA may be important in tumor angiogenesis.

In summary, this study provides new insight into the biology of FLNA and suggests that in addition to its classically known cytoskeletal function, the protein also plays an important role in the activation of ERK and AKT signaling pathways during K-RAS–induced transformation. Additionally, mice lacking *Flna* in endothelial cells developed smaller tumors. Finally, the experimental approach described here should be useful for dissecting the *in vivo* importance of *Flna* in other cancers and in tumor and physiological angiogenesis.

## Abbreviations

Ad-Cre, Adenoviral vector encoding Cre; F, Forward primer; FLNA, Human filamin A gene; Flna, Mouse filamin A gene; LSL, Floxed stop codon; MEF, Mouse embryonic fibroblasts; R, Reverse primer; VE-Cad, Vascular endothelial-specific cadherin.

## Competing interests

The authors declare that they have no competing interests.

## Authors’ contributions

RKN, MOB, LMA designed research; RKN, MXI, SB, SSN, BR, YZ performed research; RKN, MXI, AXZ, SB, DP, JB, YC, MOB, LMA analysed the data; RKN, MOB, LMA wrote the paper. All authors read and approved the final manuscript.
